# Tourism and Specific Risk Areas for *Cryptococcus gattii*, Vancouver Island, Canada

**DOI:** 10.3201/eid1411.080532

**Published:** 2008-11

**Authors:** Catharine Chambers, Laura MacDougall, Min Li, Eleni Galanis

**Affiliations:** British Columbia Centre for Disease Control, Vancouver, British Columbia, Canada (C. Chambers, L. MacDougall, M. Li, E. Galanis); University of British Columbia, Vancouver (C. Chambers, E. Galanis)

**Keywords:** Cryptococcus, disease outbreaks, travel, environmental exposure, Canada, dispatch

## Abstract

We compared travel histories of case-patients with *Cryptococcus gattii* infection during 1999–2006 to travel destinations of the general public on Vancouver Island, British Columbia, Canada. Findings validated and refined estimates of risk on the basis of place of residence and showed no spatial progression of risk areas on this island over time.

*Cryptococcus gattii* is a fungus that infects the lungs and central nervous system of mostly immunocompetent humans and animals (*1*). In 1999, *C*. *gattii* emerged on the east coast of Vancouver Island (VI), British Columbia (BC), Canada (*2*), and is now considered endemic in the environment (*3*,*4*), affecting human (*5*) and animal populations (*6*). Travel histories of patients have been used to monitor fungal spread (*5*) and to estimate the incubation period of this disease (*7**,**8*).

Intra-island travel on VI is common, and fungal exposure may not occur near residences of case-patients. Incidence rates calculated by using patient residence have suggested areas along the east coast of the island that may pose increased risk for infection (Figure) (*9*). Environmental sampling has provided evidence of the fungus over a large part of eastern VI. However, this sampling was not performed randomly and may not accurately identify areas of highest risk (*3*,*4*).

**Figure:**
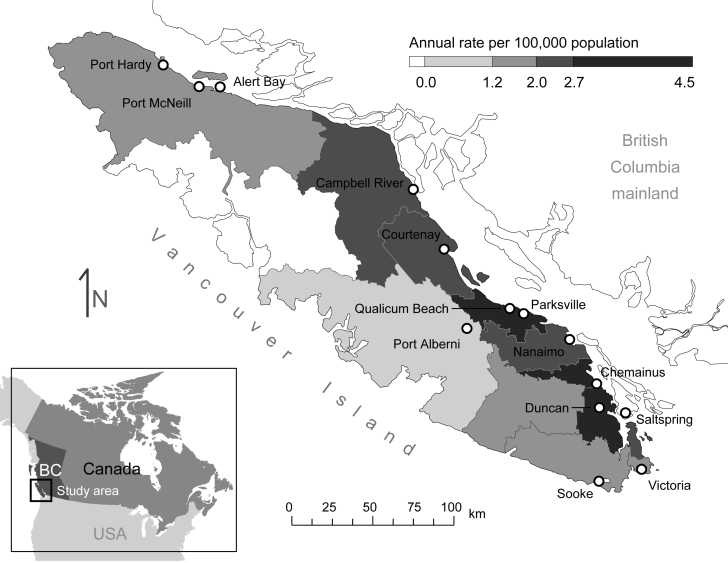
Annual rate of infection with *Cryptococcus gattii* by local administrative area, 1999–2006 (*9*), and distribution of visitor center cities on Vancouver Island, British Columbia (BC), Canada. Only visitor centers that were included in the analysis are shown.

## The Study

This study compared travel histories of *C*. *gattii*–infected case-patients with travel patterns of the general public to validate and refine these risk areas on VI. We also examined spatial progression of these areas over time to assess whether *C*. *gattii* spread from a single focal point since its emergence in 1999.

*C*. *gattii*–infected case-patients were defined as BC residents with culture-confirmed *C*. *gattii* infection or HIV-negative residents of BC with *Cryptococcus* sp. infection diagnosed by antigen detection or histopathologic analysis. Analysis included all cases diagnosed from January 1999 through December 2006 in which the patient had documented travel history on VI. Case-patients were interviewed by using a standard questionnaire and asked about travel to any city outside their city of residence in the 12 months before symptom onset or diagnosis (*8*).

Tourism BC (www.hellobc.com/en-CA/default.htm) provided aggregated monthly visitor volume to 14 visitor centers in major tourist destinations (Figure) on VI during 2000–2006. Visitors were counted if they spoke with visitor center counselors. Only visitors classified as BC residents were included in these analyses; additional personal attributes of visitors were not collected (C. Jenkins, pers. comm.). Seasonal visitor centers that had only partial data available for certain months were excluded.

Proportion of visits to each visitor center city was defined as number of visits to a visitor center city divided by total number of visits to all visitor center cities. For case-patients, the proportion was similarly defined. In both instances, visits to multiple cities by the same person were counted multiple times. Differences between proportion of case-patient visits and Tourism BC visits were evaluated by Fisher exact test and StatXact software (Cytel Inc., Cambridge, MA, USA). Analysis was conducted for all years combined and in 2 four-year increments (1999–2002 and 2003–2006) to assess potential spread of *C*. *gattii* on VI over time. Because Tourism BC visitor data were unavailable for 1999, case data for 1999–2002 were compared with aggregated Tourism BC visitor data from 2000 through 2002. Analysis was also conducted for a subset of case-patients who resided on the mainland because they represented travel exposures uncontaminated by potential exposure in place of residence. The α value for significance was adjusted to account for testing multiple visitor center cities (p = 0.05/14 visitor center cities = 0.0036). Maps were created by using ArcMap version 8.2 (Environmental Systems Research Institute Inc., Redlands, CA, USA).

Travel history data were available for 104 (60.1%) case-patients. Eighty-two (78.8%) had traveled to >1 visitor center city. Of these, 62 (75.6%) resided on VI and 20 (24.4%) lived on the BC mainland. A significantly greater proportion of visits to Parksville (18.7% vs 7.2%; p<0.0001) and Nanaimo (21.4% vs 7.4%; p<0.0001) were reported for patients than for Tourism BC visitors (Table). Similar results were obtained when analysis was restricted to earlier (1999–2002) and later (2003–2006) periods (Table).

**Table:** Proportion of cases of *Cryptococcus gattii* infection compared with proportion of BC residents who visited Tourism BC visitor centers, by location, Vancouver Island, British Columbia, Canada, 1999–2006*

Location	1999–2002				2003–2006				All years		
	No. (%) cases	No. (%) visits	% Difference		No. (%) cases	No. (%) visits	% Difference		No. (%) cases	No. (%) visits	% Difference
Nanaimo	26 (20.3)	20,160 (6.5)	13.8†		14 (23.7)	35,169 (8.0)	15.7†		40 (21.4)	55,329 (7.4)	14.0†
Parksville	25 (19.5)	24,095 (7.8)	11.8†		10 (16.9)	30,070 (6.9)	10.1		35 (18.7)	54,165 (7.2)	11.5†
Duncan	12 (9.4)	20,484 (6.6)	2.8		4 (6.8)	25,973 (5.9)	0.9		16 (8.6)	46,457 (6.2)	2.3
Victoria	24 (18.8)	58,092 (18.8)	0		16 (27.1)	94,452 (21.6)	5.6		40 (21.4)	152,544 (20.4)	1.0
Qualicum Beach	8 (6.3)	14,197 (4.6)	1.7		3 (5.1)	26,429 (6.0)	−0.9		11 (5.9)	40,626 (5.4)	0.4
Port McNeill	1 (0.8)	5,985 (1.9)	−1.2		0	6,378 (1.5)	−1.5		1 (0.5)	12,363 (1.7)	−1.1
Courtenay	8 (6.3)	35,051 (11.3)	−5.1		4 (6.8)	30,859 (7.0)	−0.3		12 (6.4)	65,910 (8.8)	−2.4
Saltspring Island	4 (3.1)	19,093 (6.2)	−3.0		1 (1.7)	20,744 (4.7)	−3.0		5 (2.7)	39,837 (5.3)	−2.7
Chemainus	3 (2.3)	13,374 (4.3)	−2.0		1 (1.7)	23,273 (5.3)	−3.6		4 (2.1)	36,647 (4.9)	−2.8
Port Alberni	4 (3.1)	23,466 (7.6)	−4.5		4 (6.8)	30,760 (7.0)	−0.2		8 (4.3)	54,226 (7.3)	−3.0
Sooke	3 (2.3)	15,450 (5.0)	−2.6		0	19,485 (4.4)	−4.4		3 (1.6)	34,935 (4.7)	−3.1
Alert Bay	0	7,891 (2.5)	−2.5		0	18,107 (4.1)	−4.1		0	25,998 (3.5)	−3.5†
Campbell River	8 (6.3)	29,219 (9.4)	−3.2		2 (3.4)	49,830 (11.4)	−8.0		10 (5.3)	79,049 (10.6)	−5.2
Port Hardy	2 (1.6)	23,106 (7.5)	−5.9		0	26,616 (6.1)	−6.1		2 (1.1)	49,722 (6.6)	−5.6†
All centers	128	309,663	–		59	438,145	–		187	747,808	–

*Visitor centers that were only opened seasonally were not included in the analysis. BC, British Columbia. †Significant differences after adjustment for multiple comparisons according to Fisher exact test (p<0.0036).

When analysis was restricted to data concerning mainland residents (patients with travel-associated exposure but no residential exposure to the fungus), a greater proportion of mainland case-patients visited Courtenay (19.4% vs 7.6%; p = 0.017), Parksville (30.6% vs 8.3%; p = 0.0001), Nanaimo (11.1% vs 6.9%; p = 0.313), and Qualicum Beach (8.3% vs 4.7%; p = 0.239) than did Tourism BC visitors during 1999–2006; however, only Parksville reached statistical significance. Because of the small number of patients who resided on the mainland (n = 20), we could not further restrict this subset analysis to earlier (1999–2002) and later (2003–2006) periods.

Residents of VI may be exposed in their place of residence, in addition to their travel destination. However, we could not accurately weight patient exposure in the home environment to exposure at the travel destination. Minor differences in results obtained for all case-patients compared with only mainland patients may be caused by this limitation or by differences in travel preferences between these groups.

Although travel history data were unavailable for 39.9% of the case-patients, they were not significantly different in terms of mean age (p = 0.303, by F test) or sex (p = 0.574, by χ^2^ test). A higher proportion of included patients resided in central VI. However, travel patterns of central VI residents did not differ from travel patterns of other VI residents (data not shown). Our analysis assumes that travel patterns of Tourism BC visitors represent those of the general BC public. However, characteristics and activities of persons who use Tourism BC visitor centers may differ from those of persons who do not. Therefore, caution is necessary when generalizing results to the entire BC population. Our interpretation is limited by its inability to account for duration of time spent in each visitor center city and specific activities of persons while there, factors that may contribute to exposure risk.

## Conclusions

Our findings suggest that the opportunity for *C*. *gattii* exposure in the areas studied has existed since the beginning of its emergence and that minimal spatial progression of risk areas has occurred over time. Areas of higher risk near Parksville and Nanaimo are consistent with distribution of environmental samples, which shows a high number of *C*. *gattii*–positive samples in these areas (*3*). Results are also consistent with annual incidence rates for *C*. *gattii* infection based on place of residence, which are highest along the central eastern coast (Figure) (*9*).

The refinement of geographic risk from our analysis may in part be the result of a reporting bias that produced larger than expected risk differences for certain areas. BC residents may be more likely to visit or travel through Nanaimo, a commercial center on VI and transportation gateway to the rest of the island (*10*), than shown in Tourism BC data. Case-patients may be more likely to report traveling to Parksville, a popular tourist destination, because it was often mentioned in media reports of the initial *C*. *gattii* outbreak. Alternatively, results may indicate a true increase in travel-associated risk in areas near Parksville and Nanaimo. Some case-patients who resided in areas with high incidence rates may have acquired their infections by travel to these 2 areas. Although Parksville and Nanaimo may represent areas of higher risk, environmental sampling suggests fungal colonization in southern and central eastern VI, and travelers can be exposed to *C*. *gattii* in these regions (*3*).

To determine travel-related risk for malaria (*11*) and gastrointestinal illness (*12*–*14*), travel patterns of case-patients have been compared with those of the general public. Use of visitor center information and tourism surveys is a cost-effective solution to derive comparison data during a retrospective investigation. This approach shows promise in assessing risk for environmental pathogens where location of exposure is unclear.
